# Genome-Wide Analysis of miR159 Gene Family and Predicted Target Genes Associated with Environmental Stress in *Dendrobium officinale*: A Bioinformatics Study

**DOI:** 10.3390/genes13071221

**Published:** 2022-07-08

**Authors:** Li Hao, Yi Zhang

**Affiliations:** 1 Institute of Fundamental and Frontier Sciences, University of Electronic Science and Technology of China, Chengdu 611731, China; haolily1991@163.com; 2 China-Croatia ‘Belt and Road’ Joint Laboratory on Biodiversity and Ecosystem Services, Chengdu Institute of Biology, Chinese Academy of Sciences, Chengdu 610041, China

**Keywords:** *Dendrobium officinale*, miR159, predicted target genes, bioinformatics, stress response

## Abstract

*Dendrobium officinale* (*D. officinale*) is a widely used traditional Chinese medicine with high economic value. MicroR159 (miR159) is an ancient and conserved microRNA (miRNA) family in land plants, playing roles in the progress of growth and development, as well as the stress response. In order to find out functions of miR159 in *D. officinale*, multiple bioinformatic approaches were employed and 10 *MIR159* genes were found, localizing on seven chromosomes and an unanchored segment of the *D. officinale* genome. All of the precursor sequences of Dof-miR159 could form a stable stem-loop structure. The phylogenetic analysis revealed that the *MIR159* genes of *D. officinale* were divided into five clades. Furthermore, the conservation analysis suggested that the 2 to 20 nt region of miR159 mature sequences were highly conserved among family members. The promoter analysis of *MIR159s* showed that the majority of the predicted *cis-*elements were related to environmental stress or hormones. In total, five classes of genes were predicted to be the target genes of Dof-miR159s, including *GAMYB transcription factors*, which had been confirmed in many other land plants. The expression patterns of predicted target genes revealed their potential roles in the growth and development of *D. officinale*, as well as in cold and drought stress responses. In conclusion, our results illustrated the stress-related miR159-targeted genes in *D. officinale*, which could provide candidate genes for resistance breeding in the future.

## 1. Introduction

MicroRNAs (miRNAs) are a class of endogenous, small noncoding RNAs of 20–24 nt in length, which are produced from longer pre-miRNAs (the precursor of microRNA) with a stem-loop structure in animals and plants. Plant miRNAs can regulate gene expression by binding mRNAs (so-called miRNA targets) through complementary sequences and inducing the degradation or translation repression [1,2,3]. Many miRNA families have been found to play essential regulatory roles in fundamental biological processes, including development, morphogenesis, and stress responses [4,5]. Benefiting from the high-throughput sequencing technology, numbers of miRNAs and the corresponding target genes have been detected in various plants, showing their functions and molecular mechanism in regulating gene expression and phenotype [6]. However, miRNA functions of relatively niche species with important ecological value still remain to be studied for utilization in traits improvement, such as medicinal plants.

Plant microRNA159 (miR159) is an ancient and highly conserved miRNA family in all dicots and monocots [7]. The *gibberellin (GA)-induced R2R3 MYB transcription factors* (named *GAMYB or GAMYB-like*) were found to be the predominant target genes of the miR159 family. In most land plants, the miR159 target sites of *GAMYBs* are highly conserved, indicating the universal regulatory relationship between miR159 and *GAMYB* among species [8]. GAMYB could bind to the GA-response elements on the promoter of downstream genes, which was first found in barley aleurone cells [9,10]. Much of the experimental evidence has shown that the miR159-*GAMYB* module is involved in plant biological processes, including seed germination [11], male sterility [12,13], and fruit development [14]. Most of these studies focused on model plants, such as Arabidopsis, rice, and tomato. In addition, the miR159 family also participated in biotic and abiotic stress response in plants. When infected by *Verticillium dahlia*, miR159 accumulated in both cotton and Arabidopsis [15]. Moreover, miR159 was found to be abundant in Arabidopsis galls after inoculation of root-knot nematodes, and the *miR159abc* triple mutant was more resistant to root-knot nematodes [16]. Drought is one of the most serious abiotic stresses that largely limits plant growth and influences yield and quality [17]. The up-regulation of miR159 was observed in many plants under drought stress, which might increase the drought tolerance of plants. By contrast, miR159 showed a decrease trend in other species with drought or salt treatment [18]. Although miR159 and its regulatory relationship with *GAMYB* are conserved among species, the roles of miR159 from different plants may be different in the stress response, which need to be further explored.

The perennial epiphytic herb *D. officinale* Kimura et Migo (*=D. catenatum* Lindl.) belongs to the largest angiosperm family, *Orchidaceae* family, and is a widely used traditional medicine [19]. *D. officinale* contains plenty of bioactive components, such as polysaccharides, alkaloids, and flavones, and has been applied to diminishing inflammation, benefiting the stomach, improving immunity, and controlling cancer [20]. In China, *D. officinale* is in enormous demand in the market due to its elegant flowers and medicinal value. However, *D. officinale* mainly distributes in subtropical mountainous regions with a warm and damp environment and is more likely to live on a stone surface or tree trunks [21]. What is more, the seeds of *D. officinale* have no endosperm and are difficult to germinate in the nature state. It was reported that the seed germination and early growth stages of *D. officinale* depended on symbiosis with specific fungi. Therefore, *D. officinale* often face long-term and various stresses during the processes of growth and development, leading to the shortage of wild resources for social needs. In recent years, artificial cultivation technology has been applied in the production of *D. officinale* while diseases are brought by high humidity, bad ventilation, and high-density planting and badly affect the yield and quality.

In this study, based on the recent released chromosome-scale assembly genome of *D. officinale* [21], transcriptome and small RNA transcriptome [22], multiple bioinformatic tools, and online websites were combined to identify Dof-miR159 gene families and the corresponding target genes. Chromosome localization and the evolution relationship of the Dof-miR159 gene family were further analyzed. Moreover, the *cis*-elements in the promoter of *Dof-MIR159s* and the expression patterns of the predicted Dof-miR159 target genes were explored, and the results suggested the potential role of the miR159-target gene module in the regulation of the development and stress response of *D. officinale*. In the future, miRNAs and their predicted target genes could be used in molecular breeding strategies for disease-resistant or stress-tolerant varieties, offering meaningful views and methods for developing the *Dendrobium* industry.

## 2. Materials and Methods

### 2.1. Identification of MIR159 Gene Family in D. officinale

Sequences of *MIR159* gene family in *D. officinale* were obtained from sRNAanno (http://www.plantsrnas.org/index.html, accessed on 5 March 2022) [23], and the chromosome-level genome of *D. officinale* was downloaded from NCBI with accession number GCA_019514585.1 (https://www.ncbi.nlm.nih.gov/assembly/GCA_019514585.1, accessed on 5 March 2022) [22].

### 2.2. Chromosome Localization of Dof-MIR159 Gene Family

In order to locate the position of *MIR159* gene family on *D. officinale* chromosome, their precursor sequences were aligned with genome of *D. officinale* using ncbi-blast-2.13.0+ (ftp://ftp.ncbi.nlm.nih.gov/blast/executables/blast+/LATEST, accessed on 15 March 2022), and the results were imported to MG2C_v2.1 (http://mg2c.iask.in/mg2c_v2.1/, accessed on 16 March 2022) for visualization.

### 2.3. Phylogenetic Analysis of Dof-MIR159s

The precursor sequences of *MIR159s* from *Arabidopsis thaliana* (L.) Heynh. (the model plant in dicots), *Oryza sativa* L. (the model plant in monocots), *Zea mays* L., and *Phalaenopsis equestris* (Schauer) Rchb.f. were downloaded from sRNAanno (http://www.plantsrnas.org/index.html, accessed on 5 March 2022) [23]. After aligned by clustal W, phylogenetic tree was conducted using Maximum Likelihood Estimate (MLE) method in MEGA 11 with a bootstrap analysis of 1000 replicates. In addition, the output file was imported into EvolView (http://www.evolgenius.info/evolview/#/treeview, accessed on 18 March 2022) for beautification.

### 2.4. Conservation Analysis of Dof-miR159 Gene Family

Multiple alignment of Dof-miR159 gene family was conducted by DNAMAN software and conservative sequences of 19 nt in length were submitted to WebLogo (http://weblogo.berkeley.edu, accessed on 19 March 2022) for base preference analysis with default parameters.

### 2.5. Structure Prediction of Dof-miR159 Precursors

The online website RNAfoldWebServer (http://rna.tbi.univie.ac.at//cgi-bin/RNAWebSuite/RNAfold.cgi, accessed on 19 March 2022) was used to predict the secondary structure of precursors of Dof-miR159 gene family.

### 2.6. Regulatory Elements Prediction on the Promoter Region of Dof-MIR159s

The online website PlantCARE (http://bioinformatics.psb.ugent.be/webtools/plantcare/html/, accessed on 20 March 2022) was employed to predict the *cis*-acting regulatory elements on the promoter (2 kbp upstream of the precursor sequence) of *Dof-MIR159s*. The number, distribution, and classification of elements were analyzed.

### 2.7. Prediction of D. officinale miR159-Targeted Genes

Target genes of Dof-miR159 were predicted using psRobot (version 1.2, created by Wu et al, Beijing, China) [24], and those with scores less than or equal to 2.0 were selected for further analysis. The relationship between Dof-miR159 and predicted target genes were visualized by Cytoscape (version 3.9.1, created by Ideker et al, San Diego, CA, USA).

### 2.8. Annotation and GO Enrichment Analysis of Predicted Target Genes in D. officinale

The online website EGGNOG-MAPPER (http://eggnog-mapper.embl.de, accessed on 22 March 2022) was employed for functional annotation of predicted target genes. At the same time, predicted target genes were also aligned to Uniprot database (https://www.uniprot.org/, accessed on 22 March 2022) using DIAMOND [25] for GO annotation. The results from EGGNOG-MAPPER and DIAMOND were merged to generate final annotation information.

### 2.9. Expression Profiling of Predicted Dof-miR159 Target Genes

The expression data of predicted Dof-miR159 target genes in different tissues were obtained from orchidbase (http://orchidbase.itps.ncku.edu.tw/est/FPKM.aspx?projectname=Dendrobium, accessed on 23 March 2022). To analyze the expression level of predicted target genes under different stresses, the transcriptome sequencing data under cold stress (SRR3210613, SRR3210621, SRR3210626, SRR3210630, SRR3210635, and SRR3210636) [26], drought stress (SRR7223298, SRR7223300, SRR7223296, and SRR7223297) [27], cadmium stress (SRR10008495, SRR10008487, SRR10008492, SRR10008497, SRR10008490, SRR10008489, SRR10008493, SRR10008494, and SRR10008499) [28], and jasmonic acid (JA) treatment (SRR14635797, SRR14635796, SRR14635793, SRR14635792, SRR14635791, and SRR14635790) [29] were downloaded from NCBI Sequence Read Archive (SRA) database. After filtering adaptor and low-quality reads by trimmomatic [30], the clean data were mapped to *D. officinale* reference genome using HISAT2 [31]. Stringtie [32] was employed to calculate Fragments Per Kilobase Million (FPKM) values to assess gene expression levels.

## 3. Results

### 3.1. Genome-Wide Identification of MIR159 Gene Family in D. officinale

Through a database of small RNAs in plants [23], we obtained 10 *MIR159* genes in *D. officinale*. The sequence and length of mature Dof-miR159s and the chromosomal locations of *Dof-MIR159s* are shown in Table 1. Most of the Dof-miR159s were 21 nt in length, while the rest were 20 nt, including Dof-miR159a, Dof-miR159c, Dof-miR159d, and Dof-miR159j. To confirm the secondary structure of the obtained precursors of the Dof-miR159s, RNAfoldWebServer (http://rna.tbi.univie.ac.at//cgi-bin/RNAWebSuite/RNAfold.cgi, accessed on 19 March 2022) was employed and all of the precursor sequences were predicted to form a stable stem-loop structure, indicating their ability to produce miRNAs (Figure S1).

**Figure 1:**
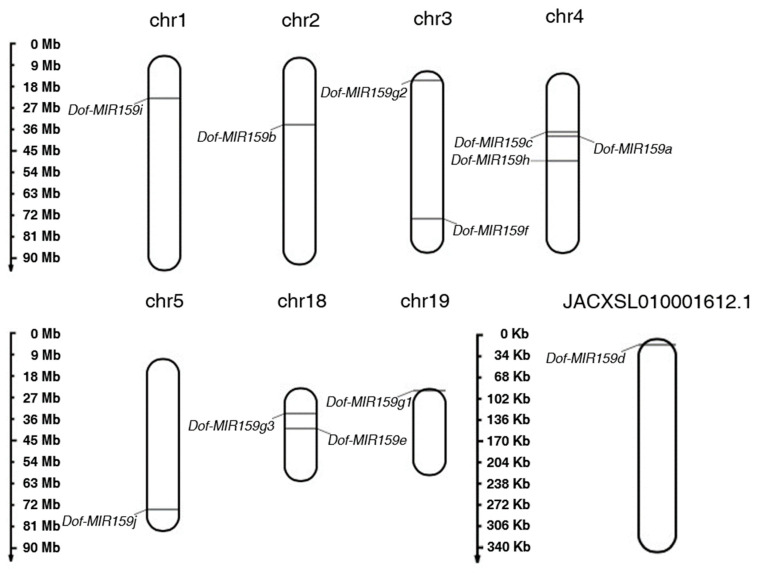
Chromosome localization of *Dof-MIR159* gene family. *Dof-MIR159* genes were localized in 7 chromosomes and an unanchored segment of *D. officinale* genome, respectively.

Based on the genome information [21], *Dof-MIR159s* were localized on seven chromosomes and an unanchored segment of *D. officinale* (Figure 1). Different from other *Dof-MIR159s*, only *Dof-MIR159g* was found to be distributed in three chromosomes, including chromosomes 3, 18, and 19 (Figure 1), which might result from gene duplication. To further investigate the conserved region in the *Dof-miR159s*, multiple alignment was conducted, and the results showed that the sequences ranging from 2 to 20 nt were relatively conserved. In addition, the base preference analysis further confirmed that 12 nt of the conserved region was completely conserved and 4 nt was relatively conserved (Figure S2).

### 3.2. Evolutionary Relationships of Dof-MIR159 Gene Family

**Figure 2:**
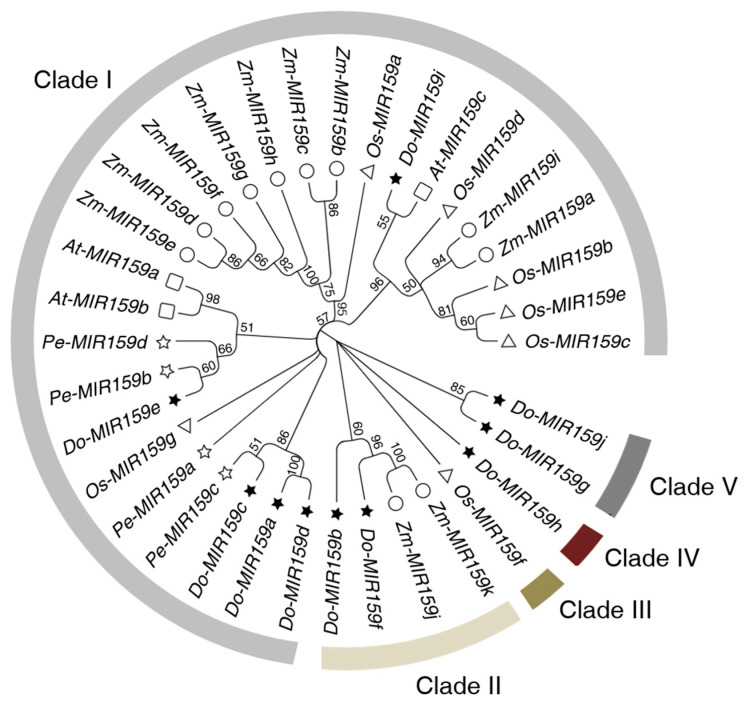
Phylogenetic analysis of *MIR159* gene of *D. officinale* and other 4 representative plants of dicots and monocots. The phylogenetic tree was constructed with 35 *MIR159* genes, using Maximum Likelihood Estimate method in MEGA 11. The bootstrap value was set as 1000 replicates. The *MIR159* genes, 3 from *A. thaliana* (*Ath-MIR159s*) marked with square, 7 from *O. sativa* (*Osa-MIR159s*) marked with triangle, 11 from *Z. mays* (*Zma-MIR159s*) marked with circle, 4 *P. equestris* (*Peq-MIR159s*) marked with hollow star, and 10 from *D. officinale* (*Dof-MIR159s*) marked with black star, were clustered into 5 clades. The light grey, buff, yellowish-brown, reddish-brown, and dark grey indicated clade I to IV, respectively.

In a previous study, *A. thaliana* was used as the model plant of dicots and *O. sativa* and *Z. mays* were representative plants of monocots in a phylogenetic tree analysis. Except the three plants mentioned above, *P. equestris*, also belonging to the *Orchidaceae* family, was also used to understand the evolutionary relationship of the *MIR159* gene family in *D. officinale.* A phylogenetic tree was constructed, following the multiple sequence alignment of 10 *D. officinale MIR159* genes, 3 *A. thaliana MIR159* genes, 7 *O. sativa MIR159* genes, 11 *Z. mays MIR159* genes, and 4 *P. equestris MIR159* genes. We found that all *MIR159* genes were divided into five clades. Half of the *Dof-MIR159*s presented were assigned to clade I, including *Dof-MIR159a*, *Dof-MIR159c*, *Dof-MIR159d*, *Dof-MIR159e*, and *Dof-MIR159i*. Most of the *Dof-MIR159* genes were clustered with the *MIR159* genes from monocots, whereas *Dof-MIR159i* was clustered with *Ath-MIR159c* (Figure 2).

### 3.3. Prediction of Cis-Acting Elements on the Promoters of the Precursor Sequences of Dof-miR159s

**Figure 3:**
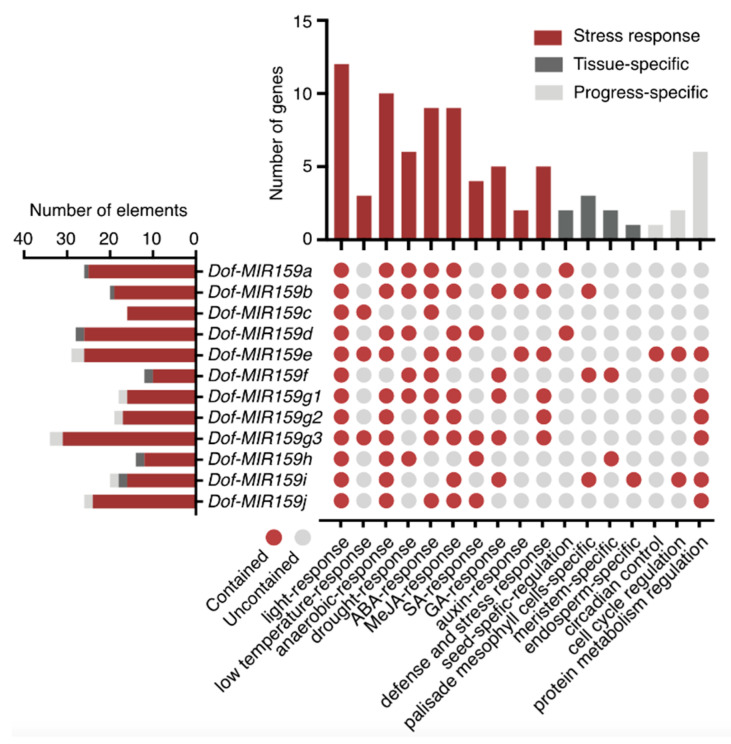
Prediction of *cis*-acting elements on the promoter of *MIR159* gene family in *D. officinale*. The image at left showed the number of *cis-*acting elements in the promoter region of *Dof-MIR159* genes. The image at middle showed the elements contained in the promoter of *Dof-MIR159* genes. The upper image showed the number of *Dof-MIR159* genes containing *cis-*acting elements related to 3 classes, including stress response (marked with red), tissue-specific (marked with dark grey), and progress-specific (marked with light grey).

To understand the possible regulatory mechanism of *Dof-MIR159s* at the transcriptional level, their promoter sequences were analyzed using the online website PlantCARE (http://bioinformatics.psb.ugent.be/webtools/plantcare/html/, accessed on 20 March 2022). All *Dof-MIR159s* contained more than 10 *cis*-acting elements, especially *Dof-MIR159g3*, which contained 34 *cis*-acting elements in its promoter (Figure 3 and Table S1). These *cis*-acting elements could be divided into three classes, including stress response, tissue-specific, and progress-specific. Notably, the majority of the elements were related to light response, anaerobic response, drought response, ABA response, MeJA response, GA response, defense and stress response, and protein metabolism regulation. Additionally, the light-response element was presented in all *Dof-MIR159* genes. The presence of these *cis-*acting elements suggested that the transcriptional expression of *Dof-MIR159s* might be predominately regulated by environmental stress or hormones.

### 3.4. Prediction of Putative Target Genes and Functional Annotation

**Figure 4:**
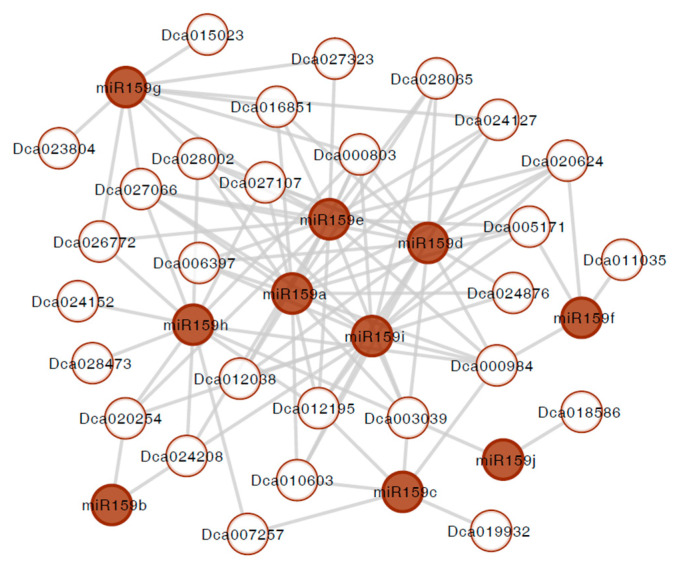
The network of Dof-miR159 gene family and their predicted target genes. The grey line between Dof-miR159s and predicted target genes represented the targeted relationship.

MicroRNAs serve functions through regulating target genes. To discover the potential function of Dof-miR159s, their target genes were predicted using psRobot_v1.2 [24]. In total, 28 predicted target genes were found and each Dof-miR159 had multiple predicted target genes. Some genes were targeted by multiple Dof-miR159s. The relationship between the Dof-miR159 gene family and their predicted target genes are visualized in the miRNA-target gene network (Figure 4).

**Figure 5:**
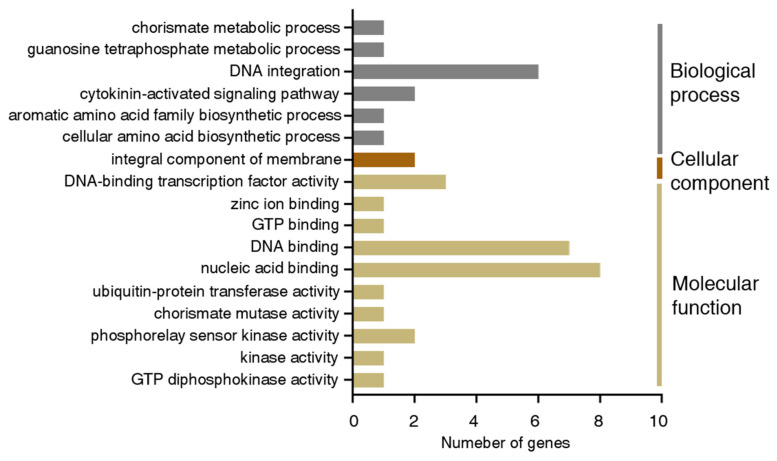
GO enrichment of predicted miR159-targeted genes in *D. officinale*. All predicted Dof-miR159-targeted genes were classified into 3 categories distinguished by different colors.

To further illustrate the roles of predicted target genes, functional annotation was conducted using the online website EGGNOG-MAPPER and Gene Ontology (GO) analysis. The results showed that the 28 predicted target genes were classified into five categories, including transcription factor *GAMYB*, *retrovirus-related Pol polyprotein from transposon retrovirus-related Pol polyprotein from transposon (RPPT) TNT 1-94*, *hypothetical protein*, *chorismate mutase 1*, and *histidine kinase CKI1* (Table S2). The most enriched GO terms of those genes were the molecular function category, which comprised DNA-binding transcription factor activity, DNA binding, nucleic acid binding, and so on. Within the biological process category, DNA integration and the cytokinin-activated signaling pathway were enriched. In addition, in the cellular component category, the integral component of the membrane was the only enriched term (Figure 5 and Table S3).

### 3.5. Tissue-Specific Expression Analysis of Predicted Dof-miR159 Target Genes

**Figure 6:**
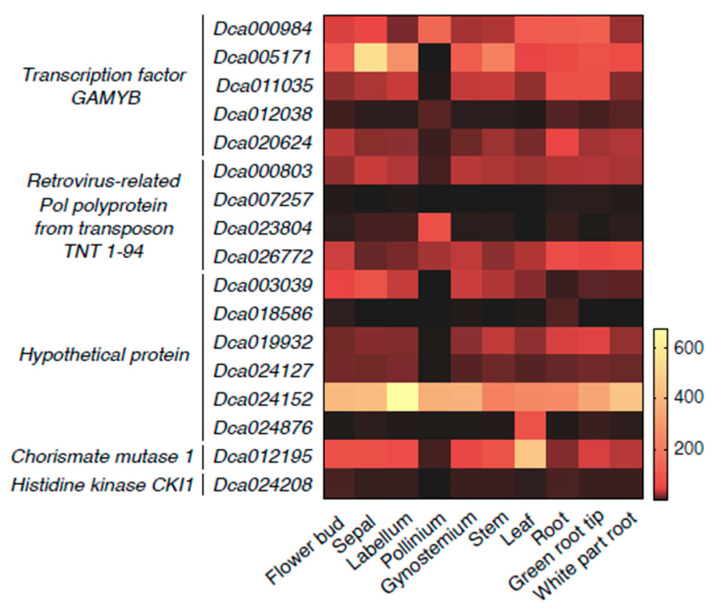
Expression patterns of predicted *D. officinale* miR159-targeted genes in different tissues. FPKM values of 28 predicted miR159-targeted genes were extracted from transcriptome sequencing data of 10 tissues in *D. officinale* using orchidbase. A total of 17 predicted miR159-targeted genes with FPKM values >1 were presented in the heat map, which was divided into 5 categories. The FPKM values ranged from 0 (black) to 600 (yellow).

Based on the above results, the predicted target genes of the Dof-miR159s were associated with diverse functions in many biological processes. We further analyzed the expression patterns of the predicted target genes in different tissues using the online website orchidbase (http://orchidbase.itps.ncku.edu.tw/est/FPKM.aspx?projectname=Dendrobium, accessed on 23 March 2022). As expected, different predicted target genes showed different expression preferences (Figure 6 and Table S4). In the class of genes encoding the GAMYB transcription factor, *Dca000984* had a higher expression level than *Dca012038* in all tissues. For other *GAMYB* genes, the expression levels in pollinium were significantly lower than in other tissues. In contrast, *Dca023804*, encoding the RPPT TNT 1-94, was highly expressed in pollinium. *Dca024208* (a member of *histidine kinase CKI1*) showed a low expression level in all tissues, while *Dca024152* (encoding the hypothetical protein) exhibited the opposite expression trend. In addition, *Dca012195* (annotated as *chorismate mutase 1*) showed a similar expression pattern with *Dca024152*, except in pollinium. These results suggested that different predicted target genes might play different roles in various tissues, indicating multiple functions of Dof-miR159s in the growth and development of *D. officinale.*

### 3.6. The Expression Pattern of Predicted Dof-miR159-Targeted Genes under Various Stresses

**Figure 7:**
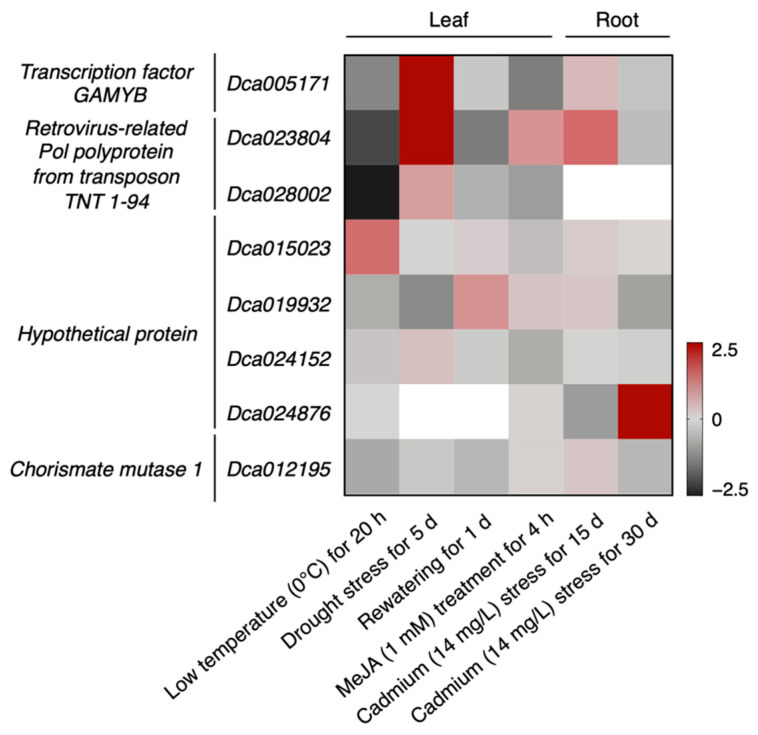
The fold change of expression level of predicted Dof-miR159-targeted genes under different stresses. The expression level of predicted miR159-targeted genes was extracted from transcriptome sequencing data of leaves under low temperature (0 °C for 20 h), drought stress (water-withholding treatment for 5 days and rewatering for 1 day), JA treatment (1 mM MeJA treatment for 4 h), and transcriptome sequencing data of roots under cadmium stress (14 mg·L^−1^ cadmium treatment for 15 and 30 days). The fold change (log_2_FC) of target genes ranged from −2.5 (black) to 2.5 (red), indicating down- and up-regulation, respectively.

To further investigate the function of predicted target genes in response to different stresses in *D. officinale,* we utilized the multiple transcriptome data of *D. officinale* under different stresses, such as low temperature [26], drought stress [27], cadmium stress [28], and JA treatment [29]. After filtrating low expression genes, eight Dof-miR159-targeted genes (FPKM > 1) were identified (Figure 7 and Table S5) and five of the eight target genes showed different expression patterns between the control and treatment (log_2_FC > 1). Both *Dca05171* (a member of the *GAMYB transcription factor*) and *Dca023804* (a member of *RPPT TNT 1-94*) were down-regulated under cold stress and up-regulated under drought stress, and *Dca05171* was also down-regulated under JA treatment. *Dca028002* (another member of *RPPT TNT 1-94*) was also down-regulated under cold stress. In the group of genes encoding the hypothetical protein, *Dca019932* was down-regulated under drought stress and *Dca024876* was up-regulated under cadmium stress. Among these differentially expressed genes (DEGs), three genes were the common targets of miR159a, miR159d, and miR159i. The two remaining genes were targeted by miR159g and miR159c, respectively. Taken together, these observations revealed that predicted miR159-targeted genes mainly participated in response to cold and drought stress.

## 4. Discussion

MicroRNAs were initially found as a group of small RNAs in animals in 1993 [33]. Compared with animals, until 2002, plant miRNAs were first identified to trigger the cleavage and degradation of internal mRNAs through complementary base pairing in Arabidopsis [34]. Over the past twenty years, numerous studies have suggested that miRNAs function as negative regulators for the expression of target genes, playing crucial roles in regulating the growth, development, and stress response in land plants.

Many miRNAs are conserved among plants, including miR159, which has been proved to participate in many fundamental biological processes. In Arabidopsis, miR159 contains three family members, including miR159a, miR159b, and miR159c, which showed small differences from each other within 1–2 nt in sequence [35]. Meanwhile, miR159s from poplar, grape, soybean, and maize harbored 3–5 nt variations [36]. In this study, 10 miR159 family members in *D. officinale* were identified (Table 1), and the sequence analysis showed that they were highly conserved, except for three sites (Figure S2). According to the phylogenetic tree, *Dof-MIR159a*, *Dof-MIR159c*, *Dof-MIR159d*, *Dof-MIR159e,* and *Dof-MIR159i* were all clustered in clade I, indicating that the precursor sequences of Dof-miR159s were relatively conserved in evolution and function. Moreover, most of the *Dof-MIR159s* were closed to those from monocotyledonous plants, which implied that miR159s of *D. officinale* might share a similar function with monocotyledonous plants. Notably, only *Dof-MIR159i* was clustered with *Ath-MIR159c*, which was from the dicot plant (Figure 2). Taken together, these observations suggested that the majority of the Dof-miR159 members were close to those from monocot plants in an evolutionary relationship, while exception also existed, which might be related to a differentiation of function.

Previous studies mainly focused on the miR159 gene family in Arabidopsis, which served as a model to study the miR159-mediated regulatory module. miR159 genes were not only found to respond to fungus, root-knot nematodes, drought stress, and salt stress but were also controlled by hormone abscisic acid (ABA) [37] and gibberellin (GA) [38]. Nowadays, the majority of the research on *D. officinale* is concentrated on bioactive ingredients, the synthesis pathway, and tissue culture. Although the high-throughput sequencing technology has been applied to medicinal plants and plenty of databases were released, the molecular mechanism of *D. officinale* in normal growth and development as well as in response to environmental stress remains to be mined. In this work, based on the prediction website, predominant *cis-*acting elements related to the stress response and phytohormone, including anaerobic response, drought response, defense and stress response, ABA response, and MeJA response, were found on the promoter of the Dof-miR159 precursors (Figure 3 and Table S1). However, the role of the Dof-miR159 gene family in biotic stress and abiotic stress remains to be further confirmed by experiments.

Arabidopsis miR159 family members were found to repress the expression of *GAMYB-like* genes (*MYB33* and *MYB65*), which induced severe impacts on the growth and phenotype of leaves and seeds [39]. It was found that miRNAs bound to target genes through complementary base pairing and one miRNA could target several genes. Similarly, one gene might be targeted by more than one miRNA. According to our results, all of the Dof-miR159 family members were predicted to have multiple target genes, among which GAMYB transcription factors associated with abiotic stress occupied a large proportion (Figure 4 and Figure 7, Tables S2 and S5). These results were consistent with previous findings [8] that *GAMYB-homologues* in most land plants harbored a highly conserved miR159 binding site. Apart from *GAMYBs*, several other predicted target genes were also found to participate in developmental and signaling pathways (Figure 5 and Figure 6, Tables S3 and S4). However, more direct evidence supporting the regulatory relationship between miRNA and predicted target genes should be provided by degradome sequencing and subsequent experiments.

Plants have developed many strategies in rapid response to environmental stress by producing small signaling molecules, such as inorganic ions [40], small RNAs [41], and hormones [42]. MiRNAs are small RNAs with a short length, which have been well-studied and developed into molecular biology tools to improve plant traits in stress-resistance breeding [43,44], while it lags behind in the field of Chinese medicine. In this work, we focused on the predicted miRNA-targeted genes in response to stress and five DEGs were found to respond to cold, drought, cadmium stress, and JA treatment. Among these DEGs, *Dca005171*, down-regulated under cold stress and JA treatment (Figure 7 and Table S5), was completely aligned to the transcription factor *GAMYB* (LOC110114505), using blastn (https://blast.ncbi.nlm.nih.gov/Blast.cgi?PROGRAM=blastn&PAGE_TYPE=BlastSearch&LINK_LOC=blasthome, accessed on 28 March 2022) of the Basic Local Alignment Search Tool (BLAST) in the NCBI. The promoter of *GAMYB* (LOC110114505) contained multiple hormone-related *cis*-acting elements, including the ARE, ABRE, GARE-motif, and TCA-element. Moreover, this gene was reported to be down-regulated in response to cold stress [45], which is consistent with our results. However, *Dca005171* expression was significantly induced under the water-withholding treatment for 5 days and decreased after rewatering (Figure 7 and Table S5). These findings suggested that *GAMYB* genes could be differently regulated under different stresses, which might result from the expression change of corresponding miRNA159s. Most proteins can be functionally annotated via an experimental and computational approach, but some proteins with unknown domains are referred to as hypothetical proteins [46]. *Dca019932*, one of the predicted Dof-miR159-targeted genes encoding the hypothetical protein, was enriched in terms of DNA-binding transcription factor activity by the GO analysis (Table S3). Moreover, *Dca019932* expression significantly decreased under the water-withholding treatment for 5 days and increased after rewatering for 1 day (Figure 7 and Table S5), indicating its potential role in transcriptional regulation under drought stress. Intriguingly, *Dca024876* (encoding another hypothetical protein) showed an opposite expression trend under cadmium stress for 15 and 30 days (Table S5). We assumed that the expression of *Dca024876* was not only regulated by cadmium stress but also affected by plant conditions. However, the functions of those predicted genes in response to stress still need to be validated via RT-qPCR and subsequent experiments. In previous studies, the *RPPT* gene was identified to be related to DNA methylation and contributed to phenotypic plasticity in response to environmental stress [47]. We observed that the expression levels of *Dca023804* and *Dca028002* (members of *RPPT TNT 1-94*) were regulated under both cold and drought stress (Figure 7 and Table S5), suggesting their potential functions in abiotic stress. Whether they could lead to phenotypic changes and the mechanisms behind that should be illustrated in the future.

Notably, *Dca005171*, *Dca024876,* and *Dca028002* were all predicted target genes of Dof-miR159a/d/i, and in that case, it is worth verifying whether we can successfully regulate multiple genes and improve several resistances at the same time through editing one single Dof-miR159.

## 5. Conclusions

Based on bioinformatics, we identified the miR159 gene family and the corresponding predicted target genes in response to environmental stress in *D. officinale* for the first time, which needs further experimental validation. Importantly, this study not only enriches our understanding of the miR159-target gene network under different stresses but also provides multiple candidates for traits improvement in the resistance breeding of *D. officinale*.

## Figures and Tables

**Table 1:** Information on miR159 gene family in *D. officinale*.

Dof-miRNA159 Gene Family	Mature Sequences (5′-3′)	Length (nt)	Chromosome Localization
Dof-miR159a	UUUGGAUUGAAGGGAGUUCC	20	chr4: 27722223–27721971
Dof-miR159b	GUUGGAUUGAAGUGAGCUCUG	21	chr2: 29534166–29533917
Dof-miR159c	UUUGGCUUGAAGGGAGCUCC	20	chr4: 25802668–25802903
Dof-miR159d	UUUGGAUUGAAGGGAGUUCC	20	JACXSL010001612.1: 9402–9654
Dof-miR159e	UUUGGAUUGAAGGGAGCUCUG	21	chr18: 17853945–17854139
Dof-miR159f	UUGGAGUGAAGGGAGCUCCAU	21	chr3: 65301674–65301356
Dof-miR159g	UUUGGAUUGAAGGAAGUUCUG	21	chr19: 647221–647302
			chr3: 4033924–4033843
			chr18: 10991639–10991558
Dof-miR159h	UUUGGGUUGAAGGGAGCUCUG	21	chr4: 38860800–38860456
Dof-miR159i	CUUGGAUUGAAGGGAGCUCC	21	chr1: 18800452–18800688
Dof-miR159j	UUGGGUUUGAAGGGAGCUCUA	20	chr5: 66058489–66058730

## Data Availability

The sequences of MIR159s from *A. thaliana*, *O. sativa*, *Z. mays*, *P. equestris*, and *D. officinale* were obtained from sRNAanno (http://www.plantsrnas.org/index.html, accessed on 5 March 2022) [23], and the chromosome-level genome of *D. officinale* was downloaded from the NCBI with the accession number GCA_019514585.1 (https://www.ncbi.nlm.nih.gov/assembly/GCA_019514585.1, accessed on 5 March 2022) [22]. The expression data of the predicted Dof-miR159-target genes in different tissues were obtained from orchidbase (http://orchidbase.itps.ncku.edu.tw/est/FPKM.aspx?projectname=Dendrobium, accessed on 23 March 2022). The transcriptome sequencing data under cold stress (SRR3210613, SRR3210621, SRR3210626, SRR3210630, SRR3210635, and SRR3210636) [26], drought stress (SRR7223298, SRR7223300, SRR7223296, and SRR7223297) [27], cadmium stress (SRR10008495, SRR10008487, SRR10008492, SRR10008497, SRR10008490, SRR10008489, SRR10008493, SRR10008494, and SRR10008499) [28], and jasmonic acid (JA) treatment (SRR14635797, SRR14635796, SRR14635793, SRR14635792, SRR14635791, and SRR14635790) [29] were downloaded from the NCBI Sequence Read Archive (SRA) database (https://www.ncbi.nlm.nih.gov/sra/, accessed on 10 March 2022).

## Supplementary Materials

The following supporting information can be downloaded at: https://www.mdpi.com/article/10.3390/genes13071221/s1, Figure S1: Predicted secondary structure of Dof-miR159 precursors. The mature sequence of Dof-miR159s was highlighted with red. Figure S2: The conserved domain analysis of Dof-miR159s. Table S1: Summary information of predicted *cis*-acting elements in the promoter of *Dof-MIR159* gene family. Table S2: Functional annotation of predicted miR159-targeted genes in *D. officinale*. Table S3: GO enrichment of the 28 predicted target genes of Dof-miR159s. Table S4: FPKM values of the 17 predicted targeted genes (FPKM > 1) in different tissues. Table S5: FPKM values of 8 *D. officinale* predicted miR159-targeted genes (FPKM > 1) under different stresses.

## Abbreviations

miRNA	microRNA
nt	Nucleotide
RPPT	retrovirus-related Pol polyprotein from transposon
DEGs	differentially expressed genes
