# Supplementary figures and images for: Genome-Wide Analysis of miR159 Gene Family and Predicted Target Genes Associated with Environmental Stress in Dendrobium officinale: A Bioinformatics Study
Source: Genes (Basel). 2022 Jul 8;13(7):1221. doi: 10.3390/genes13071221 (PMC9320484; doi:10.3390/genes13071221)

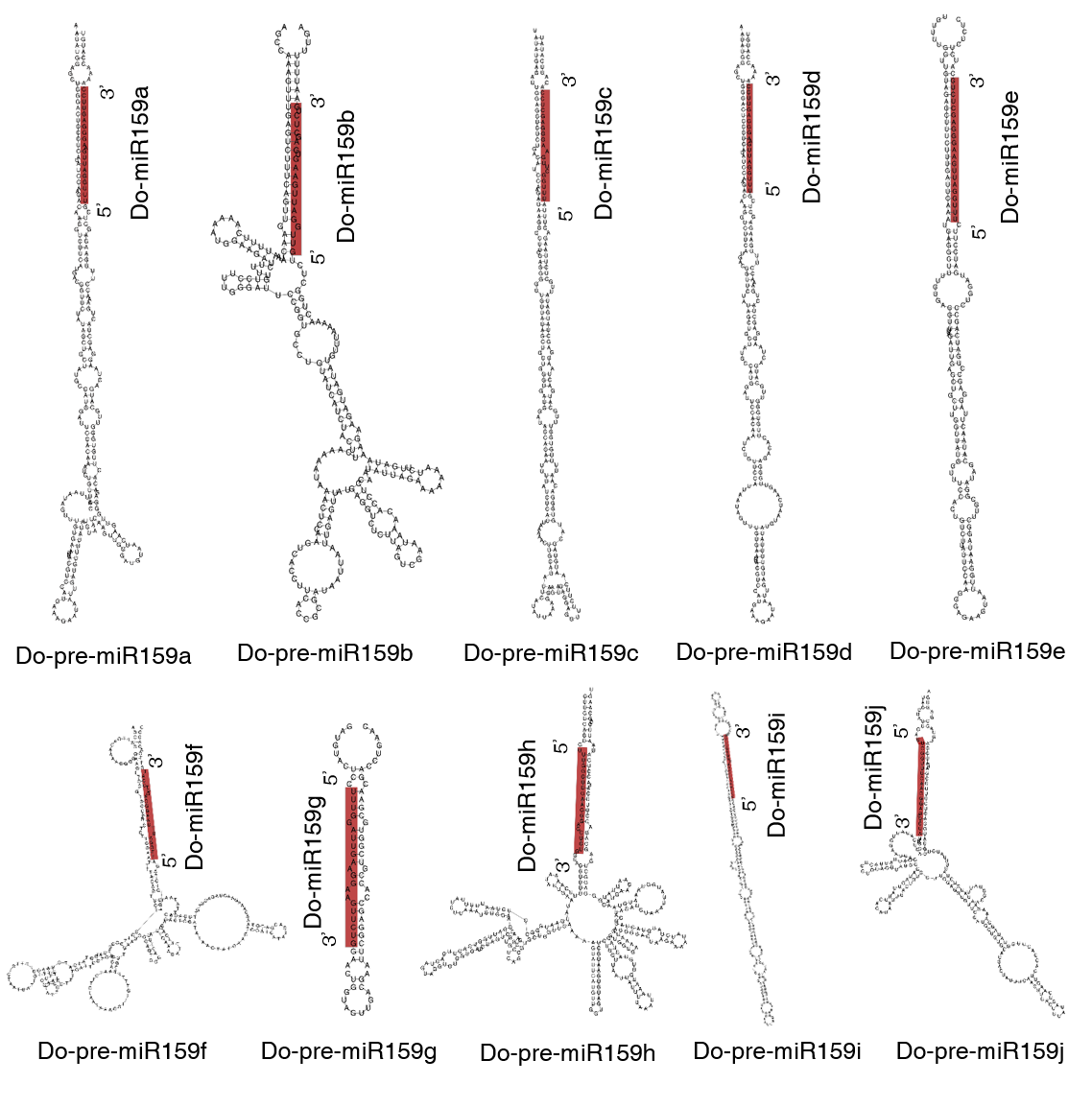

Supplement: Supplementary file 1 [file genes-13-01221-s001.zip › Figure S1-revised.png]

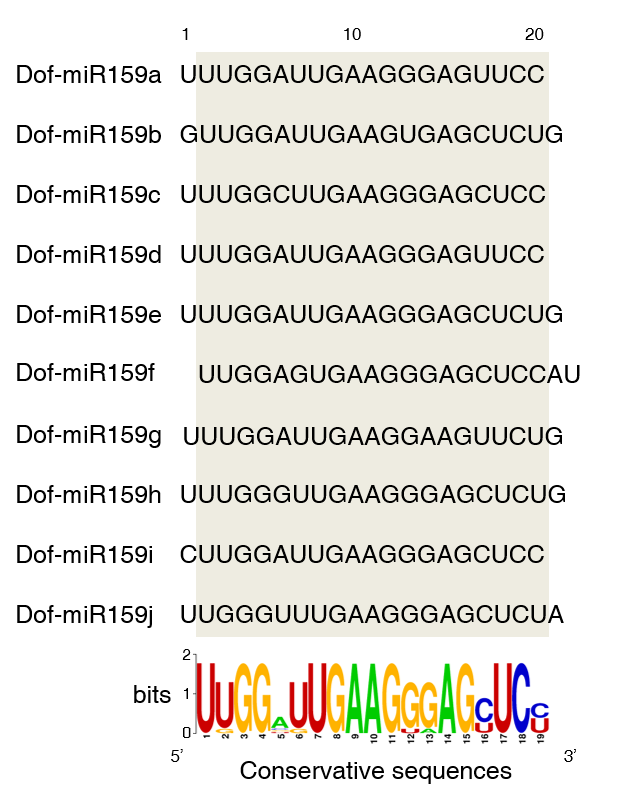

Supplement: Supplementary file 1 [file genes-13-01221-s001.zip › Figure S2-revised.png]
